# Probing the early stages of shock-induced chondritic meteorite formation at the mesoscale

**DOI:** 10.1038/srep45206

**Published:** 2017-05-30

**Authors:** Michael E. Rutherford, David J. Chapman, James G. Derrick, Jack R. W. Patten, Philip A. Bland, Alexander Rack, Gareth S. Collins, Daniel E. Eakins

**Affiliations:** 1 Institute of Shock Physics, Blackett Laboratory, Imperial College London, London SW7 2BW, UK; 2 Department of Earth Science and Engineering, Imperial College London, London SW7 2BP, UK; 3 Department of Applied Geology, Curtin University of Technology, Perth, WA 6845, Australia; 4 European Synchrotron Radiation Facility, Structure of Materials, Grenoble, France

## Abstract

Chondritic meteorites are fragments of asteroids, the building blocks of planets, that retain a record of primordial processes. Important in their early evolution was impact-driven lithification, where a porous mixture of millimetre-scale chondrule inclusions and sub-micrometre dust was compacted into rock. In this Article, the shock compression of analogue precursor chondrite material was probed using state of the art dynamic X-ray radiography. Spatially-resolved shock and particle velocities, and shock front thicknesses were extracted directly from the radiographs, representing a greatly enhanced scope of data than could be measured in surface-based studies. A statistical interpretation of the measured velocities showed that mean values were in good agreement with those predicted using continuum-level modelling and mixture theory. However, the distribution and evolution of wave velocities and wavefront thicknesses were observed to be intimately linked to the mesoscopic structure of the sample. This Article provides the first detailed experimental insight into the distribution of extreme states within a shocked powder mixture, and represents the first mesoscopic validation of leading theories concerning the variation in extreme pressure-temperature states during the formation of primordial planetary bodies.

Probing the properties of granular materials under extreme conditions is a key step in understanding how primordial dusty aggregates were lithified to form the rocky building blocks of the solar system^1^,^2^,^3^, and the efficacy of proposed asteroid deflection techniques^4^. More generally, understanding how the inherent properties of granular materials (initial porosity, grain morphology) may be tailored to produce a desired bulk performance or encourage/discourage reactions is of both fundamental and industrial scientific interest^5^,^6^.

Traditionally, the dynamic compression response of porous materials has been probed via surface-level diagnostics such as VISAR^7^. While these experiments have been used to provide information on the equilibrated shock state in various materials and have allowed for a calibration of continuum level models^8^, they inherently struggle to resolve the time-evolution of dynamic compaction at the sub-mm scale within the material. As a result these techniques are limited in their ability to discern exactly which mesoscopic processes govern material deformation at the macroscopic scale, and thus provide data which is not optimal for validating mesoscopic models^9^. Very recently, a number of *in-situ*, X-ray radiography experiments have been performed on shock-compressed materials at synchrotron light sources and X-ray free electron lasers. These studies have provided quantitative measurements of wave speed^6^, density^10^, three-dimensional sample morphology^11^, and instability growth^12^ with sufficient spatiotemporal resolution to resolve the first stages of shock-compression at the mesoscale. For example, the temporal evolution of spatially-resolved volumetric density behind an elastic shock front has been measured^10^. To date, however, high spatiotemporal resolution synchrotron X-ray radiography has not been applied to the study of dynamically loaded granular systems in detail.

Low velocity impacts were recently proposed as a mechanism for the compaction and lithification of primordial high porosity, loosely aggregated planetesimals^2^, a key step in the formation of rocky planetary bodies. Chondritic meteorites (chondrites), the most common meteorite type found on Earth, represent the surviving remnants of their primitive parent bodies and provide a record of early solar system processes. They are composed of mm-scale, dense spherical inclusions (chondrules), surrounded by a matrix of micrometre-sized grains. Chondrule abundance, matrix porosity and bulk porosity vary between individual samples and groups, but experimental^1^ and observational^2^,^13^ evidence suggests that the precursor material was a mixture of chondrules and a highly distended matrix of finer-grained particles (matrix porosity: 60–80%; bulk porosity: 30–50%).

Impact compaction of chondrites was investigated experimentally on the cm-scale by Beitz *et al*.^1^ who performed impact experiments on a range of precursor chondrite simulants (initial bulk porosities 70–31%) at approximate stresses of <2 GPa. X-ray tomography was used to quantify the final porosity in recovered samples, which revealed that these modest stresses were sufficient to yield final porosities in line with those of real chondrites. A number of numerical studies^2^,^3^ have extended these conclusions by examining the shock compression process through mesoscopic modelling in the iSALE^14^,^15^ hydrocode. These simulations, which modelled the matrix as a continuum and explicitly tracked deformation within each chondrule corroborated the experimental measurements of final porosity and also allowed for a more detailed examination of how the compaction process occurs. It was observed that the dynamic densification and related temperature rise is almost entirely undertaken by the porous matrix with the chondrules acting as near bystanders. For example, Bland *et al*.^2^ report a large difference in the post-shock matrix (>800 K) and chondrule (~70 K) temperature changes. These numerical observations are consistent with observations in real chondrites which show temperature excursions and deformation within matrix material adjacent to unscathed chondrules^2^,^13^,^16^ and a highly-complex response to shock compaction^17^, highlighting the usefulness of probing shock-compression in granular materials at a mesoscopic level.

In this Article, the study of shock-compressed analogue precursor chondrite material using *in-situ*, single-bunch phase-contrast X-ray radiography at the European Synchrotron Radiation Facility (ESRF) is presented. These experiments, which represent the first impact loading studies at ESRF and the first detailed synchrotron X-ray radiography experiments on shock-compressed granular systems, allowed measurements of spatially-resolved wave velocities and wavefront thickness as a function of time. As well as providing mesoscale, *in-situ* experimental data to complement the post-situ experiments and mesoscale modelling already discussed, the data presented here represent a step-change in how the shock compaction of porous materials may be understood in terms of a distribution of shock states rather than spatially-averaged metrics.

## Method

**Figure 1:**
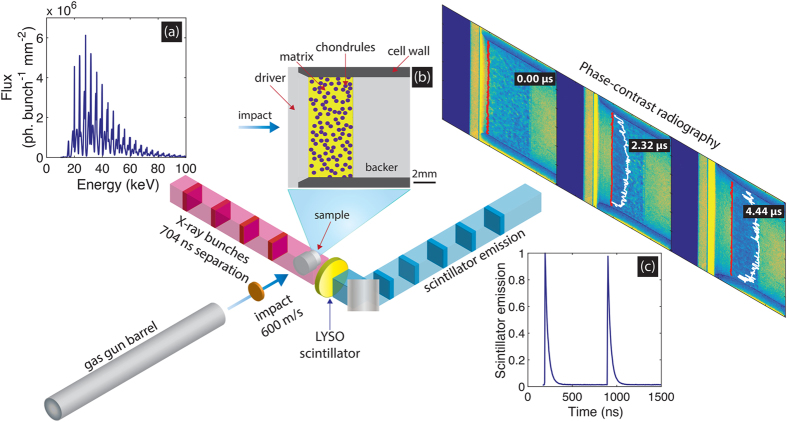
Illustration of the dynamic X-ray radiography experiments. Samples were shock compressed by flyer plate impact at ~600 ms^−1^. Material deformation was examined *in-situ* using single bunch, hard X-ray phase-contrast radiography transverse to the impact direction. Two radiographs were recorded per shot. The X-ray radiography was synchronised to the impact process via pre-impact light gates and a train of triggers in-phase with the X-ray bunches delivered by Beamline ID19. (**a**) Spectral flux per bunch through an on-axis 1 mm^2^ area delivered by Beamline ID19 in the four bunch mode (40 mA storage ring current), including 2.8 mm diamond and 1.4 mm aluminium filtering. The total flux was 1.2 × 10^9^ photons s^−1^ mm^−2^ on-axis. (**b**) Illustration of the target geometry. A bimodal powder mixture was contained in a cylindrical aluminium cell, sealed on the impact surface by a polycarbonate driver plate and the rear surface by a polycarbonate backer. (**c**) Results of a decay scan measurement of the emission from LYSO:Ce as a function of time, showing that the bunch structure is well-resolved with a negligible background between bunches^39^.

Experiments were performed on Beamline ID19 at ESRF^18^. Figure 1 provides an illustration of the dynamic radiography experiments. A single stage light-gas gun^19^ with a 12.7 mm bore was installed on the beamline perpendicular to the X-ray beam. In this work, for each bimodal mixture (large and small chondrule simulants) two dynamic stress states were explored through polycarbonate (630 ± 4 ms^−1^) or copper (596 ± 3 ms^−1^) flyer plate impact. Polycarbonate sabots were used in both cases. At these impact velocities the expected initial bulk sample stresses were in the range of interest for chondrite formation; 0.56 Gpa (Polycarbonate flyer) to 1.21 GPa (copper flyer)^20^,^21^. These stresses were estimated using impedance matching with a calculated Hugoniot. The calculation of the mixture Hugoniot is described in Section *Mixture Hugoniot calculations* in Supplementary Information.

**Figure 2:**
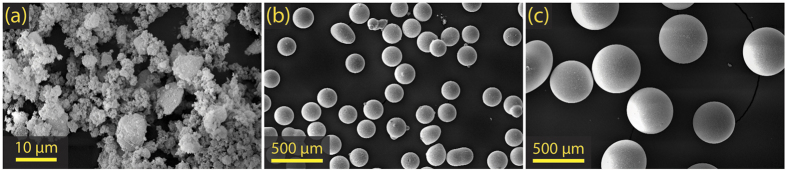
Scanning electron micrographs of (**a**) sipernat 320-DS powder, (**b**) soda-lime microspheres (196 μm mean diameter and (**c**) soda-lime microspheres (425 μm mean diameter). Data were recorded with a JSM5610LV scanning electron microscope at 20 kV.

Impact experiments were performed on bimodal powder mixtures analogous to precursor chondrite material^13^. Samples consisted of soda-lime microspheres, acting as chondrule simulants, of either 196 μm (small chondrule) or 425 μm (large chondrule) mean diameter (GP0196 and GP0425 Whitehouse Scientific, UK), dispersed throughout a porous silica matrix. Sipernat 320-DS (Sipernat) amorphous silica was chosen as the matrix analogue due to its small grain size (≲10 μm), which allowed for a matrix/guest grain size ratio on the order of 100:1, similar to real chondrites. Scanning electron micrographs of the powders are shown in Fig. 2, which show the sipernat grains to be formed of several smaller (~1 μm) grains with varying intragranular porosity, and the chondrule simulants to be spherical. The solid densities of the sipernat and soda-lime spheres were 2.2 gcm^−3^ and 2.46 gcm^−3^, respectively. Powder samples were prepared with volume fractions within the range of inferred chondrite precursor proportions: 30% volume fraction soda-lime spheres, 70% volume sipernat matrix, 74% sipernat matrix porosity. Samples were contained in 10 mm diameter, 11 mm tall, 1 mm thick aluminium cells. The impact surface of the cell was sealed with a 2 mm thick polycarbonate driver plate. The rear surface of the cell was sealed with a 10 mm thick polycarbonate backer. Samples were pressed to a final thickness of 4.4 mm, yielding a matrix density of 26% solid density. Figure 1(b) shows an illustration of the powder cell. For simplicity, shots performed with a copper flyer will be referred to as Cu-PC (copper flyer:polycarbonate driver) or high-stress, and those performed with a polycarbonate flyer will be referred to as PC-PC (polycarbonate flyer:polycarbonate driver) or low-stress.

Single-bunch, transmission phase-contrast^22^ X-ray radiography was used to diagnose shock-induced sample deformation. Figure 1(a) shows the calculated on-axis spectral X-ray flux (1.2 × 10^9^ photons per bunch mm^−2^) delivered per bunch (150 ps duration^23^) on Beamline ID19 in the four bunch mode. The four bunch mode at ESRF delivered an X-ray bunch every 704 ns, which was well-suited to stroboscopically probing the gas gun experiments over several microseconds. Unlike other dynamic phase contrast radiography installations^10^,^24^ Beamline ID19 can deliver a beam size of over 100 mm^2^, which allowed for the entire powder bed to be radiographed and thus offered the chance to observe physics evolve from the micrometre to millimetre spatial scales, and to probe a distribution of shock states. Two X-ray radiographs were recorded on each gas gun shot via a 200 μm thick, 25 mm diameter LYSO:Ce, Ca single-crystal scintillator (Crystal Photonics, FL, USA) coupled to a Princeton Instruments PI-MAX4:1024i intensified CCD (ICCD) camera. The ICCD recorded a field of view of 12.1 × 12.1 mm. A lower bound (worst case) on the system spatial resolution of 71 μm ± 12 μm (6 ± 1 pixels) was determined from measurements of the system edge-spread-function. The ICCD exposure time was 650 ns and was synchronised so as to record the scintillator emission from one X-ray bunch. Synchronisation of the X-ray radiography with the gas gun experiments and additional details of the X-ray detection method are discussed in Section *X-ray detection and synchronisation* in Supplementary Information.

### Data availability

The experimental data presented in this Article are available open access at: https://doi.org/10.5281/zenodo.159474.

## Results

**Figure 3:**
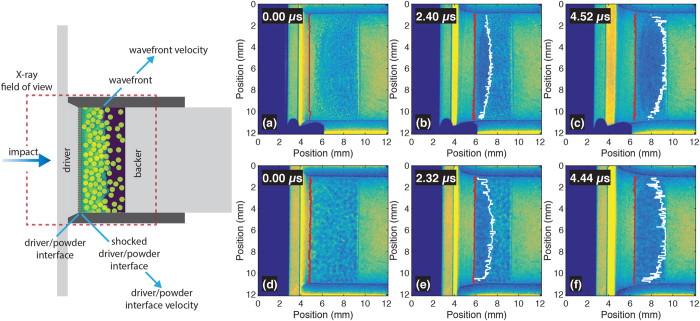
Representative single-bunch radiographs recorded on both mixture types. (Left): To-scale illustration of the impact experiments with impact occurring from left to right. The approximate radiography field of view is shown as a red dotted line. (Top row): Radiographs recorded on a small chondrule mixture experiment. (**a**) Pre-shot radiograph with the driver/powder interface shown in red. (**b**) and (**c**) Radiographs recorded 2.40 μs and 4.52 μs after the shock wave arrived at the driver/powder interface, respectively. The shocked driver/powder interface is shown in red and the shock front is shown in white. (Bottom row): Radiographs recorded on a large chondrule mixture experiment. (**d**) Pre-shot radiograph. (**e**) and (**f**) Radiographs recorded 2.32 μs and 4.44 μs after the shock wave arrived at the driver/powder interface, respectively. Interfaces are marked similarly to the small chondrule radiographs. In both cases a blank polycarboante sabot impacted the polycarbonate driver plate at 633 ± 23 ms^−1^.

A total of 7 shock experiments are reported here with two dynamic radiographs recorded per shot. Figure 3 shows a representative series of radiographs recorded from two shots, one on small chondrule mixtures and one on large chondrule mixture (one pre-shot and two dynamic radiographs).

Radiographs were corrected for dark frame noise, and flatfield correction was performed to correct the spatially inhomogeneous illumination imparted by the Gaussian-shaped undulator beam. Flatfield radiographs were scaled according to the storage ring current (which varied significantly in the 4 bunch mode) in order to maintain quantitative grey levels. Depletion of charge in the ICCD intensifier microchannel plate (MCP)^25^ led to a reduction in gray level of the highest-count features in the second radiograph recorded on each shot. These effects were corrected by calculating frame1-frame2 intensity relationships for every pixel on the CCD and rescaling the gray levels in the second frame.

To extract spatially-resolved wave speeds from the radiographs a number of interface detection routines were employed. Edge detection with Canny’s method^26^ reliably detected the cell wall/powder interfaces. The driver/powder interfaces were detected on every row of pixels either via fitting a step function to the gray level contrast across the interface or estimating the edge position via the observed phase-contrast fringe. In both cases, the width of the defining feature (step or fringe) was taken as the error and was typically on the order of 10 pixels (119 μm). The wavefront position was detected by difference imaging, which calculated the first pixels whose gray levels were statistically different (lower than one standard deviation from the mean) from a clearly unshocked region. The difference imaging method measured an upper bound on the wavefront position in the radiographs and the wavefront position error was set to the system spatial resolution of 6 pixels (71 μm). It could not be assumed that waves were steady in the powder bed. Accordingly, wavefront velocity was calculated differently for the first and second radiographs recorded on each shot. In the first frame, the wavefront distance was calculated by subtracting the static driver/powder interface position from the wavefront position. This was converted to wavefront velocity (*U*_*wf*_) by dividing the result by the calculated transit time in the powder bed. The calculation of this transit time required impedance matching at the flyer/driver and driver/powder interfaces. Hugoniot data for polycarbonate and copper were obtained from the LASL Shock Hugoniot Data Compendium^27^. For the second frame, the wavefront transit distance was calculated as the difference in wavefront positions between the two radiographs and the transit time was equal to the interframe time. Driver/powder interface velocities (*U*_*dp*_) were calculated similarly from the measured driver/powder interface positions.

**Figure 4:**
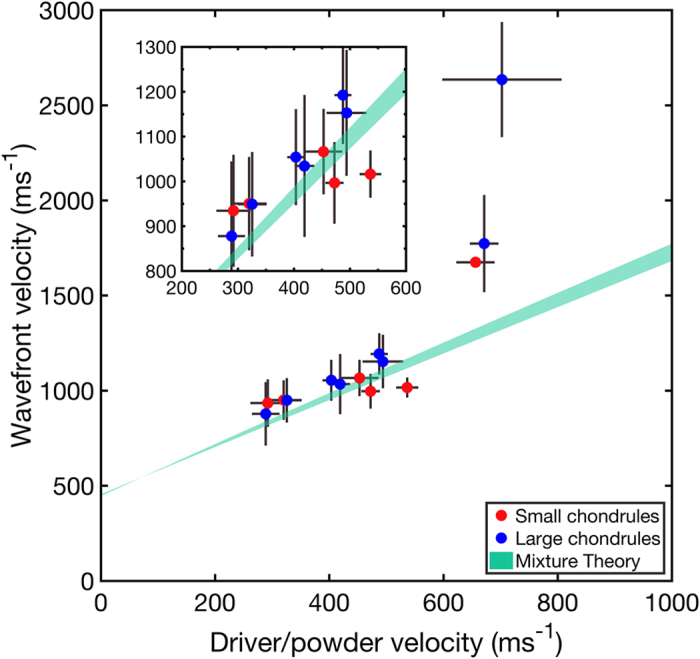
Measured on-axis (*U*_*wf*_, *U*_*dp*_) pairs, determined as the mean shock/particle velocities observed in a centred 10-chondrule-diameter region of the radiographs. Error bars represent +/− 2 standard deviations from the mean in the on-axis region. Small and large chondrule mixture data are shown by red and blue markers, respectively. A calculated band of Hugoniot states representing bimodal mixtures with initial matrix densities spanning 24% to 29% solid density is shown in teal. (Inset): A zoomed in region between *U*_*dp*_ = 200–600 ms^−1^.

Figure 4 shows on-axis wavefront velocity (*U*_*wf*_), driver/powder interface velocity (*U*_*dp*_) states measured directly from the radiographs. To obtain these values the wave velocities in centred 10-chondrule-diameter regions of the radiographs were binned according to Scott’s rule^28^, which assumed the data were normally distributed. The size of these regions was 1.960 mm and 4.250 mm for the small and large chondrules, respectively. The data sets were well-fitted by a normal distribution, from which a mean velocity and standard deviation were derived. The data shown in Fig. 4 are therefore the mean velocities in the on-axis region with error bounds of +/− two standard deviations, which was chosen to capture the microstructure-dominated distribution of velocities observed. Also shown in Fig. 4 is a calculated band of shock states. These Hugoniot states were calculated using the *ε*−*α* model^15^,^29^, and the mixture theory proposed by Batsanov^30^. The range of states was generated by varying the initial bulk sample density within the error bounds of the balance used to weigh-out the powder component mass (±0.01 g). These states thus represent average initial matrix densities spanning 24% to 29% solid density.

## Discussion

In the driver/powder interface velocity range of 200 ms^−1^–600 ms^−1^ the measured on-axis wavefront speeds for small and large chondrule mixtures were the same within error (mean ± standard deviation); small: 979 ± 58 ms^−1^, large: 1043 ± 119 ms^−1^. This suggests that the macroscopic shock response is governed by bulk sample composition and is relatively insensitive to the constituent size, in agreement with previous impact experiments^31^,^32^.

Assuming that the initial on-axis density was equal to the bulk density (1.09 g cm^−2^) the mean bulk stress and post-shock porosity in the chondrite mixtures was estimated to be 0.45 ± 0.13 GPa and 20 ± 9%, respectively. These values are consistent with previous numerical simulations of impact compaction of primitive meteoritic material^2^ confirming the applicability of this work to understanding compaction in real chondrites.

The velocity error bounds determined via fitting a normal distribution to the on-axis regions are larger than those derived from interface detection methods. Moreover, the error in wavefront velocity is larger, approximately 2-fold, for large chondrule mixtures. These observations corroborate the velocity error bounds being representative of a distribution of microstructure-dependent shock states, and confirms that the leading-edge wavefront position measured via the difference imaging method can extract mesoscopic information about the shock process. The relative error in the driver/powder interface velocities is similar for both chondrule simulant sizes, suggesting that the approach taken to locate this interface (via significant absorption or phase contrast changes) was more representative of a spatially-averaged measurement of material velocity. The limiting factor in detecting spatially-resolved interfaces in the radiographs was low contrast-to-noise ratios (<1 in some cases) as a result of noise introduced by the PI-MAX4:1024i intensifier^25^. Future work should consider improvements to the methodology in order to limit the amount of intensification needed.

Three on-axis (*U*_*wf*_, *U*_*dp*_) pairs at *U*_*dp*_ > 600 ms^−1^ were observed, which appear to be outliers. The observed outlier velocities were compared with impedance matching calculations. Driver/powder interface velocities higher than the impact velocity can only be achieved by the shocked polycarbonate driver releasing into a material of lower impedance, such as the porous matrix. However, such a scenario would result in shock velocities on the order of 1100 ms^−1^ (*ε*−*α* calculations predict *U*_*s*_ = 1134 ms^−1^ at *U*_*p*_ = 600 ms^−1^). Therefore, it is proposed that these outlier states are disconnected measures of wavefront and driver/powder interface velocity in which the radiographic method has detected a wavefront velocity dominated by chondrule-chondrule stress-bridges^33^. This conclusion is corroborated by observations of the initial sample configuration on these shots which appeared to be chondrule-dense at the driver/powder interface. More generally, it was assumed in comparing the mixture theory calculation with the on-axis data that the material composition (volume fraction and matrix porosity) in the on-axis regions was equivalent to the bulk density calculated from sample preparation methods. A higher bulk density in the on-axis region would contribute to higher wavefront velocities. Future work could consider alternative measures of material velocity behind the wavefront, perhaps through the use of displacement correlation methods. Furthermore, X-ray tomography could be used to provide a three-dimensional characterisation of the initial sample configuration prior to impact in order to better understand the origin of anomalous velocities and variations in sample composition. It was also noted that these three outlier states were the earliest-time (after the wave entered the powder bed) data recorded and as such, they were most susceptible to systematic uncertainties in calculations of time-after-impact, which relied upon impedance-matching calculations of wave transit time in the driver. Future work could improve on this by fielding more robust triggers to determine more precisely when the wave entered the powder bed.

In order to capture the effect of release on the measured shock states radiographs were recorded at times spanning 0.35 μs–4.94 μs transit time in the powder bed. The leading lateral release speeds were estimated using the method of characteristics^34^ assuming the calculated Hugoniot at 20% bulk porosity approximated the isentrope of the shocked material. These release speeds were 2770 ms^−1^ (polycarbonate flyer, 0.56 GPa impact pressure) and 3490 ms^−1^ (copper flyer, 1.21 GPa impact pressure), respectively. The time at which the leading lateral release waves reached the shock front on-axis in the PC-PC and Cu-PC loadings were estimated to be 3.30 μs and 2.53 μs, respectively. In the Cu-PC shots it was estimated that longitudinal release waves arrived at the driver/powder interface after 1.23 μs and reached the shock front after 1.59 μs. It was therefore acknowledged that some of the states presented in Fig. 4 are partially released. To ascertain with confidence which points represent Hugoniot and release states, however, requires a more careful consideration of the release process and in turn, a more rigorous understanding of the initial sample density. This could be readily achieved via X-ray tomography and should be a key focus of future work. Within the scope of this Article it was concluded that the shocked and partially released states populate similar regions in velocity space.

**Figure 5:**
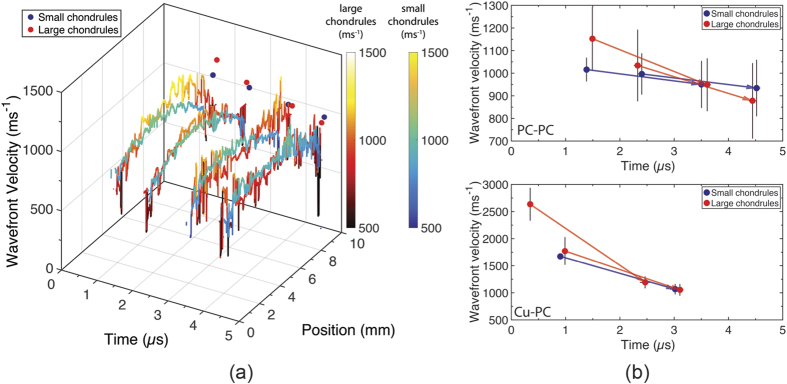
(**a**) Spatially-resolved wavefronts as a function of time for small and large chondrule mixtures impacted with a polycarbonate flyer. Colormaps and colorbars indicate wavefront velocity magnitude: small chondrules (blue/green/yellow), large chondrules (black/red/yellow). In both cases, the on-axis shock velocities as a function of time are plotted on the rear axes. Wave front curvature is seen to decrease as a function of time. The on-axis values show a gradually decreasing wave speed over the duration of the experiment. These spatially-resolved lineouts emphasise the diversity in shock states that exist within a powder bed. (**b**) On-axis shock velocities as a function of time for chondrule samples impacted with a polycarbonate flyer (upper figure) and copper flyer (lower figure). Arrows connect pairs of data points (one datum for each radiograph) measured on the same shot and thus, the same initial sample configuration. Therefore, the PC-PC figure contains data from four shots and the Cu-PC figure contains data from three shots. A larger initial shock velocity that decreased more quickly over time was observed in the large chondrule mixtures. It should be re-emphasised that while the error bars appear relatively large they represent 4*σ* of the normally-distributed shock states in the powder mixtures.

Spatially resolved measurements of wave speed not only reveal a distribution of shock states within the material but also permit insight into the influence of sample composition and release on wavefront thickness and curvature. Figure 5(a) shows spatially-resolved shock fronts as a function of time for PC-PC shots alongside the on-axis shock speed values. Significant heterogeneity was observed in the wavefronts on top of the curvature introduced by lateral release. In keeping with the estimation that some of the measured (*U*_*wf*_, *U*_*dp*_) states were released it was observed that shock velocity decreased as a function of time. These observations are promising for the validation of release models, and when combined with measurements of wavefront curvature suggest that mesoscale measurements of shock front dispersion and perturbation decay could be made to probe the effective viscosity of granular materials^9^. In Fig. 5(b) the on-axis wave speeds are shown with arrows connecting measurements made on the same shot and initial sample configuration for Cu-PC and PC-PC shots. These velocity data show that at early times large chondrule mixtures achieved initially higher shock velocities and subsequently achieved velocities similar to those reached by PC-PC shots. The quicker velocity decrease observed in large chondrule mixtures (Fig. 5(b)) in combination with an apparently higher shock/particle velocity slope in large chondrule mixtures (Fig. 4) could be indicative of stress-bridge dominated, microstructure-dependent release behaviour from the incompletely compacted (20% porous) state.

**Figure 6:**
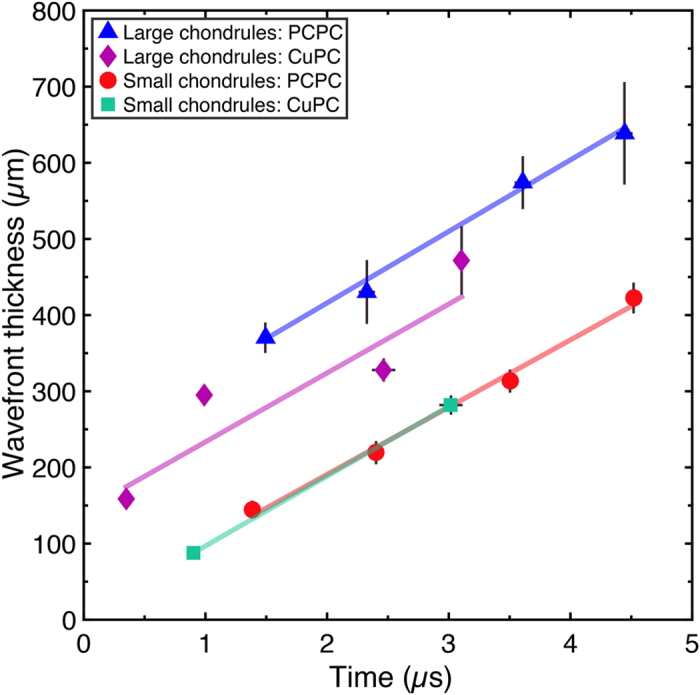
Wavefront thickness as a function of time for both sample mixtures and loading conditions. Blue triangles: Large chondrule mixtures impacted with a polycarbonate flyer, Purple diamonds: Large chondrule mixtures impacted with a copper flyer, Red circles: Small chondrule mixtures impacted with a polycarbonate flyer, Teal squares: Small chondrule mixtures impacted with a copper flyer. Linear fits to each data set are also shown. All data sets showed a similar rate of wavefront thickness increase over time. In most cases the markers occlude the timing error bars.

Figure 6 shows wavefront thickness as a function of time for both mixtures and loading conditions. The radiographic intensity in the shocked region of the powder was not constant and an average intensity representative of the shocked state could therefore not be reliably determined. As a result, the measure of wavefront thickness presented here represents the spatial extent of the leading portion of the wavefront. Details of the wavefront thickness calculation are presented in Section *Wavefront thickness calculations* in Supplementary Information. Table 1 shows the rates at which wavefront thickness increased.

The largest wavefront thicknesses were seen in the lower stress PC-PC impacts on large chondrule mixtures. These values (370–639 μm) are approximately double those seen in both the small chondrule mixture shots (88–422 μm), with the high-stress Cu-PC impacts on large chondrule mixtures producing intermediate values. Both mixtures showed wavefront thicknesses on the order of 1–2 chondrule diameters. These observations are in agreement with previous studies^31^,^35^,^36^, which show a reduction in transmitted shock wave rise-time at finer particle sizes and higher input stresses, the clear advancement here being that the current measurements need not be corrected for interactions with the backer. The ability to make such direct measurements of wavefront dispersion emphasises again how this technique represents an improved method for measuring the release behaviour and effective viscosity of shocked granular systems^9^. The large magnitude of errors in the rate of wavefront thickness increase (13–78%) are interpreted physically as being representative of the variation in sample preparation and chondrule packing, and the subsequent collective treatment of several samples. This again emphasises the influence of the initial sample configuration on the compaction process. Assuming that each chondrule size and loading condition can be treated as a contiguous data set it was observed that the rate of wavefront thickness increase or dispersion over time was similar in all four cases, which could be attributed to the common matrix properties of both mixtures; while the absolute thickness of the wavefront is linked to the sample chondrule size. More experiments are required at larger post-impact times to determine if wavefront thicknesses tend towards an integer number of grain diameters as seen in 2D mesoscopic modelling^2^,^37^,^38^. Additionally, later-time observations would help to discriminate between microstructure-dependent effects and the possible obscuration of the true wavefront thickness by the radiographic method which necessarily integrates through the sample thickness.

## Summary

In summary, the shock-compression of analogue precursor chondrite material was studied *in-situ* with high-spatiotemporal resolution X-ray phase-contrast radiography. A number of wave velocity pairs were measured directly from the radiographs, which were in good agreement with continuum-level predictions. The measured wave velocities were combined with the jump conditions to confirm that the low shock states achieved in this work compacted the analogue precursor meteorite material to a final bulk porosity consistent with observed porosities in ‘unshocked’^20^,^21^ meteorite samples.

The data presented here show that within a heterogeneous granular sample there is a distribution of thermodynamic states, which appear to be highly-sensitive to the initial material configuration and intimately linked to the sample microstructure. Furthermore, this Article has presented experimental insight into the temporal evolution of these states and how their mean values and statistical error bounds may be reconciled in terms of traditional measures of shock-compression such as shock velocity, particle velocity pairs.

To properly understand the distribution of states and shock variables obtained during the dynamic loading of a granular system requires a rigorous understanding of its initial geometry. Future work will consider the use of X-ray tomography to both characterise the initial sample state for the interpretation of experimental data and provide realistic sample states for mesoscale simulations. With a refined understanding of thermodynamic state distributions through experiment and validated simulations it may be possible to draw conclusions on the distribution of pressure and temperature states within a shocked chondrite mixture, which will go some way to understanding the relative abundance of high-pressure-temperature phases found in recovered chondrites today.

## Additional Information

**How to cite this article:** Rutherford, M. E. *et al*. Probing the early stages of shock-induced chondritic meteorite formation at the mesoscale. *Sci. Rep.* **7**, 45206; doi: 10.1038/srep45206 (2017).

**Publisher's note:** Springer Nature remains neutral with regard to jurisdictional claims in published maps and institutional affiliations.

## Supplementary Material

Supplementary Information

## Figures and Tables

**Table 1:** Rate of wavefront thickness increase.

Chondrule Size	Mean Chondrule Size (μm)	Loading condition	Rate of wavefront thickness increase (μm μs^−1^)
Small	196	PC-PC	88 ± 11
Small	196	Cu-PC	92 ± 0
Large	425	PC-PC	94 ± 11
Large	425	Cu-PC	90 ± 70
