# Corrigendum: Probing the early stages of shock-induced chondritic meteorite formation at the mesoscale

**DOI:** 10.1038/srep46912

**Published:** 2017-12-22

**Authors:** Michael E. Rutherford, David J. Chapman, James G. Derrick, Jack R. W. Patten, Philip A. Bland, Alexander Rack, Gareth S. Collins, Daniel E. Eakins

Scientific Reports 7: Article number: 45206; 10.1038/srep45206 published online: 05 30 2017; updated: 12 22 2017.

This Article contains errors. In the legend of Figure 1,

“(**a**) Spectral flux per bunch through an on-axis 1 mm^2^ area delivered by Beamline ID19 in the four bunch mode (40 mA storage ring current), including 2.8 mm diamond and 1.4 mm aluminium filtering. The total flux was 1.2 × 10^9^ photons s^−1^ mm^−2^ on-axis.”

should read:

“(**a**) Spectral flux per bunch through an on-axis 1 mm^2^ area delivered by Beamline ID19 in the four bunch mode (40 mA storage ring current), including 2.8 mm diamond and 1.4 mm aluminium filtering. The total flux was 4 × 10^7^ photons/bunch/mm^2^ on-axis.”

In the Methods section,

“Figure 1(a) shows the calculated on-axis spectral X-ray flux (1.2 × 10^9^ photons per bunch mm^−2^) delivered per bunch (150 ps duration^23^) on Beamline ID19 in the four bunch mode.”

should read:

“Figure 1(a) shows the calculated on-axis spectral X-ray flux (4 × 10^7^ photons/bunch/mm^2^ on-axis) delivered per bunch (150 ps duration^23^) on Beamline ID19 in the four bunch mode.”

In the Discussion section,

“The time at which the leading lateral release waves reached the shock front on-axis in the PC-PC and Cu-PC loadings were estimated to be 3.30 μs and 2.53 μs, respectively.”

should read:

“The times at which the leading lateral release waves reached the shock front on-axis in the PC-PC and Cu-PC loadings were estimated to be 2.3 μs and 1.9 μs, respectively.”

