# Supplementary material for: Probing the early stages of shock-induced chondritic meteorite formation at the mesoscale
Source: Sci Rep. 2017 May 30;7:45206. doi: 10.1038/srep45206 (PMC5448141; doi:10.1038/srep45206)
Supplement: Supplementary Information [file srep45206-s1.pdf]

Supplementary Information: Probing the early stages of shock-induced chondritic meteorite formation at the mesoscale

Michael E. Rutherford[1,*], David J. Chapman[1], James G. Derrick[2], Jack R.W. Patten[1], Phil A. Bland[3], Alexander Rack[4], Gareth S. Collins[2], and Daniel E. Eakins[1]

[1]Institute of Shock Physics, Blackett Laboratory, Imperial College London, London SW7 2BW, UK
[2]Department of Earth Science and Engineering, Imperial College London, London SW7 2BP, UK
[3]Department of Applied Geology, Curtin University of Technology, Perth, WA 6845, Australia
[4]European Synchrotron Radiation Facility, Structure of Materials, Grenoble, France
[*]m.rutherford13@imperial.ac.uk

ABSTRACT

Chondritic meteorites are fragments of asteroids, the building blocks of planets, that retain a record of primordial processes. Important in their early evolution was impact-driven lithification, where a porous mixture of millimetre-scale chondrule inclusions and sub-micrometre dust was compacted into rock. In this Article, the shock compression of analogue precursor chondrite material was probed using state of the art dynamic X-ray radiography. Spatially-resolved shock and particle velocities, and shock front thicknesses were extracted directly from the radiographs, representing a greatly enhanced scope of data than could be measured in surface-based studies. A statistical interpretation of the measured velocities showed that mean values were in good agreement with those predicted using continuum-level modelling and mixture theory. However, the distribution and evolution of wave velocities and wavefront thicknesses were observed to be intimately linked to the mesoscopic structure of the sample. This Article provides the first detailed experimental insight into the distribution of extreme states within a shocked powder mixture, and represents the first mesoscopic validation of leading theories concerning the variation in extreme pressure-temperature states during the formation of primordial planetary bodies.

Supplementary Information

This document provides supplementary information for the article *Probing the early stages of shock-induced chondritic meteorite formation at the mesoscale*.

X-ray detection and synchronisation

This Section provides additional details of the X-ray detection method and explains how single-bunch radiographs were recorded during the gas gun experiments.

Phase-contrast was introduced to the radiographs via a source-sample distance of 150 m (which delivered a wave-front with partial spatial coherence) and a sample-scintillator propagation distance of 9067 ± 25 mm. This propagation distance was within the near-field limit[1] of 13.6 m at 30 keV, assuming two-pixel resolution. The scintillator emission was relayed to the ICCD with a 1.08X, NA = 0.3 optical relay composed of back-to-back Hasselblad HC2.2 100 mm lenses operating at infinity. This resolution was assumed to be constant across the field of view. This assumption was corroborated by the MTF curves provided for the HC2.2 100 mm lenses provided by Hasselblad, which report a variation in contrast of $< 5\%$ over the 12.1 mm image height at spatial frequencies of up to 40 lp/mm with NA 0.23. Figure **??(c)** shows a bunch structure scan[2] with the same LYSO:Ce,Ca scintillator. Due to the single-mode, fast decay (τ = 41 ns) of LYSO:Ce,Ca the bunch structure was clearly resolved[2] and the scintillator emission decreased to zero, within the statistics of the dark frame noise, between bunches. Accordingly, dynamic radiographs did not suffer from ghosting artefacts. Using the Dual Image Feature of the PI-MAX4:1024i ICCD two radiographs were recorded per experiment with a typical interframe time of 2112 ns. This interframe time, equal to three bunch separations, was limited by the decay time of P46 (YAG:Ce)[2] used in the PI-MAX4:1024i intensifier. The spectral flux delivered by Beamline ID19 was well-suited to transmission experiments on the silica-based samples with, for example, 35% transmission at 30 keV through 10 mm of solid SiO_2.

Synchronisation of the plate-impact experiments with the synchrotron radiography was achieved via the radio-frequency (RF) bunch clock and projectile light gates in a similar manner to that described previously.[3] Briefly, the gas gun firing triggered a fast beamline shutter, which remained open for 200 ms, greater than the typical ~ 170 ms duration of the entire experimental process. This shutter was in place to minimise heat load to the sample and scintillator. Prior to impact the projectile interrupted two optical light gates (19.28 ± 0.02 mm (LG1) and 7.3 ± 0.02 mm (LG2) from the impact surface), which allowed for an on-shot measurement of impact velocity with a typical error of 5%. The LG2 signal was recorded on a logic oscilloscope (Tektronix DPO7254), which also recorded a constant train of signals in phase with the X-ray bunches. These signals were provided by the Bunch Clock Delay Unit installed on Beamline ID19. After the LG2 signal fell to 50% of its initial intensity a trigger was sent to the ICCD camera upon receipt of the next X-ray bunch signal. A radiograph was then recorded after a time determined prior to the experiment. This trigger-to-radiograph time was calculated in advance as the sum of the projectile transit time between LG2 and the impact surface (calculated through the measured LG2-impact distance and expected projectile velocity and impedance matching at the projectile/driver interface). The maximum impact-to-radiograph jitter was therefore 704 ns, limited by the storage ring bunch mode. The second radiograph was recorded at a pre-determined time after the first, typically 2112 ns in this study. The ICCD monitor signals were recorded on the same oscilloscope as the X-ray trigger train and the falling LG2 signal, which allowed for an accurate measurement of the impact-to-radiograph time with, again, errors on the order of 5%. Finally, the shock transit time through the driver plate was subtracted from the impact-to-radiograph times to yield the time at which the radiograph was recorded after the wave had entered the powder bed. Errors in the measured inter-lightgate distances and timing signals were propagated in quadrature.

Mixture Hugoniot calculations

Considering the range of values used to calculate the equation of state of fused silica (e.g. the range in reported Grüneison constants: 0.012[4], 0.036[5] and 0.9[6]) the relatively simple approach to calculating the shock properties of a mixture proposed by Batsanov[7] was preferred to more complex methods such as the popular 0K-isotherm method by McQueen.[8]

For a given shock pressure the mixture particle velocity was calculated as the root-mean-square of the component particle velocities, averaged by their mass fractions. This is shown by Eq. 1.

$$U_{p,mix}(P) = \sqrt{\mu_{SL} \cdot u_{p,SL}(P)^2 + \mu_{FS} \cdot u_{p,FS}(P)^2}, \tag{1}$$

where $U_{p,mix}(P)$ is the mixture particle velocity at a pressure P, $u_p(P)$ is the particle velocity of a component at pressure P, and μ is the mass fraction of the component. The subscript SL refers to solid soda-lime and FS to porous fused silica.

The associated shock velocity for the mixture was calculated using Eq. 2 with a volume-weighted initial density of $1.09\,\mathrm{g\,cm^{-3}}$, calculated using Eq. 3.

$$U_{s,mix}(P) = \frac{P}{\rho_{0,mix} \cdot u_{p,mix}(P)}, \tag{2}$$

where $U_{s,mix}(P)$ is the mixture shock velocity at a pressure P.

$$\rho_{0,mix} = \nu_{SL} \cdot \rho_{0,SL} + \nu_{FS} \cdot \rho_{0,FS}, \tag{3}$$

where $\rho_{0,mix}$ is the mixture initial density, and ν is the initial volume fraction of the mixture component.

The component Hugoniots for soda-lime and sipernat (fused silica) were calculated using a Mie-Gruneison equation of state. The influence of initial porosity in the fused silica Hugoniot was accounted for using the ε-α model.[9,10] Table 1 shows the parameters used in the calculation. The ε-α parameters α_x, κ and χ were varied to provide the best fit to plate-impact data determined previously for sipernat.[11]

Table 1. Equation of state and $\varepsilon - \alpha$ compaction model parameters.

Material	Grüneisen constant	Initial Density ($\mathrm{g\,cm^{-3}}$)	α_0	α_x	κ	χ
Soda Lime	0.14[12]	2.53	-	-	-	-
Fused Silica	0.036[5]	2.20	3.85	1.29	0.8	0.33

Wavefront thickness calculation

This Section provides details of how wavefront thicknesses were calculated.

A statistical measure of wavefront thickness was sought to complement the statistical analysis of velocity data. Binning the raw wavefront position data was likely to overestimate the magnitude of wavefront thickness due to presence of curvature induced by lateral release. Therefore, a 200 point moving average was subtracted from each wavefront vector, which yielded a nominally flat wavefront. The flattened wavefront positions were then binned according to Scott's rule[13] and fitted with a normal distribution. The fitted normal distribution was relatively insensitive to the number of points including in the preceding moving average. Wavefront thickness was then taken as four standard deviations of the fitted distribution; the same measure which was taken as the error bound on the on-axis velocity data. The error in the wavefront thickness was taken as four times the error in the fitted value of the Gaussian standard deviation (derived from the least-squares fitting routine). The rate and error at which wavefront thickness was increasing were determined from an error-weighted least-squares linear fit to the data.

References

1. Weitkamp, T., Haas, D., Wegrzynek, D. & Rack, A. ANKAphase: software for single-distance phase retrieval from inline X-ray phase-contrast radiographs. *J. Synchrotron Rad.* **18**, 617–629 (2011).
2. Rutherford, M. E. *et al.* Evaluating scintillator performance in time-resolved hard X-ray studies at synchrotron light sources. *J. Synchrotron. Radiat.* **23**, 685–693 (2016).
3. Eakins, D. E. & Chapman, D. J. X-ray imaging of subsurface dynamics in high-Z materials at the Diamond Light Source. *Rev. Sci. Instrum.* **85**, 123708–12 (2014).
4. Stacey, F. D. . *Physics of the Earth* 303–305 (1992).
5. Wackerle, J. Shock-Wave Compression of Quartz. *J. Appl. Phys.* **33**, 922–17 (1962).

6. Borg, J. P. *et al.* Computational simulations of the dynamic compaction of porous media. *Int. J. Impact Eng.* **33**, 109–118 (2006).

7. Batsanov, S. S. *Effects of Explosions on Materials: Modification and Synthesis Under High-Pressure Shock Compression*, 1–45 (Springer New York, New York, NY, 1994).

8. McQueen, R. G., Marsh, S. P., Taylor, J. F., Fritz, J. N. & Carter, W. J. The equation of state of solids from shock wave studies. In Kinslow, R. (ed.) *High-Velocity Impact Phenomena*, 293 – 417 (Academic Press, 1970).

9. Wünnemann, K., Collins, G. S. & Melosh, H. J. A strain-based porosity model for use in hydrocode simulations of impacts and implications for transient crater growth in porous targets. *Icarus* **180**, 514–527 (2006).

10. Collins, G. S., Melosh, H. J. & Wünnemann, K. Improvements to the *epsilon*-α; porous compaction model for simulating impacts into high-porosity solar system objects. *Int. J. Impact Eng.* **38**, 434–439 (2011).

11. Chapman, D. J. *Shock-compression of porous materials and diagnostic development*. Ph.D. thesis, University of Cambridge (2010).

12. Dulieu-Smith, J. M. & Stanley, P. On the interpretation and significance of the Grüneisen parameter in thermoelastic stress analysis. *J. Mater. Proc. Technol.* **78**, 75–83 (1998).

13. Scott, D. W. On optimal and data-based histograms. *Biometrika* **66**, 605–610 (1979).
